# The influence of procedural volume on short-term outcomes for robotic pancreatoduodenectomy—a cohort study and a learning curve analysis

**DOI:** 10.1007/s00464-023-09941-8

**Published:** 2023-03-08

**Authors:** Michal Kawka, Tamara M. H. Gall, Fiona Hand, Scarlet Nazarian, David Cunningham, David Nicol, Long R. Jiao

**Affiliations:** 1 Department of Medicine, Imperial College London, London, UK; 2 Department of Academic Surgery and Cancer, The Royal Marsden Hospital, 203 Fulham Rd, London, SW3 6JJ UK; 3 Department of Surgery and Cancer, Imperial College London, London, UK

**Keywords:** Robotic surgery, Pancreatoduodenectomy, Learning curve, Surgical outcomes

## Abstract

**Background:**

An increasing number of robotic pancreatoduodenectomies (RPD) are reported, however, questions remain on the number of procedures needed for gaining technical proficiency in RPD. Therefore, we aimed to assess the influence of procedure volume on short-term RPD outcomes and assess the learning curve effect.

**Methods:**

A retrospective review of consecutive RPD cases was undertaken. Non-adjusted cumulative sum (CUSUM) analysis was performed to identify the procedure volume threshold, following which before-threshold and after-threshold outcomes were compared.

**Results:**

Since May 2017, 60 patients had undergone an RPD at our institution. The median operative time was 360 min (IQR 302.25–442 min). CUSUM analysis of operative time identified 21 cases as proficiency threshold, indicated by curve inflexion. Median operative time was significantly shorter after the threshold of 21 cases (470 vs 320 min, *p* < 0.001). No significant difference was found between before- and after-threshold groups in major Clavien-Dindo complications (23.8 vs 25.6%, *p* = 0.876).

**Conclusions:**

A decrease in operative time after 21 RPD cases suggests a threshold of technical proficiency potentially associated with an initial adjustment to new instrumentation, port placement and standardisation of operative step sequence. RPD can be safely performed by surgeons with prior laparoscopic surgery experience.

**Supplementary Information:**

The online version contains supplementary material available at 10.1007/s00464-023-09941-8.

## Introduction

Pancreatoduodenectomy (PD) remains a mainstay curative approach for head and neck of pancreatic, periampullary and duodenal, and distal bile duct tumours [1]. Pancreatoduodenectomy, which was first described in 1898, is a technically challenging procedure, requiring multiple dissection and reconstruction steps, for which traditionally an open surgical approach (OPD) is utilised [2]. However, with the increased recognition of benefits of minimally invasive surgical techniques, such as decreased blood loss, decreased length of hospital stay, and better functional outcomes, laparoscopic (LPD) and robotic pancreatoduodenectomy (RPD) have been established as potential alternatives. Yet, while adoption of minimally invasive techniques has been ubiquitous for procedures such as cholecystectomy and appendicectomy, the uptake for complex hepatopancreatobiliary procedures has been slow due to concerns over technical complexity, as well as morbidity and mortality outcomes [3, 4].

These concerns revolve around the learning curve for new surgical techniques, as it was previously shown in LPD that as many as 104 cases might be needed before expert proficiency [5]. As such, the consensus on the equivalence or potential superiority of LPD to OPD has not been reached, with two randomised controlled trials showing no differences in morbidity and mortality between the approaches [6, 7], but a third one being terminated early due to high mortality in the LPD group [8]. What is more, concerns over high conversion rates, reaching nearly 25%, were raised, confirming the technical challenges of LPD [7].

As such, similar questions are being posed regarding RPD, particularly given the current higher cost of robotic surgery tools. However, robotic surgery may address some of the technical difficulties of LPD, providing 3D vision with increased depth perception, additional degrees of freedom due to ‘endo-wristed’ instruments, standard instruments for robotic operation and eliminating both instrument and camera tremor and variability [9]. Indeed, the learning curve for basic surgical skills is significantly less with a robotic compared to laparoscopic platform [10]. Therefore, in surgery requiring complex technical skills such as PD, the use of robotic systems could help with dissection and reconstruction precision and reduce surgeon fatigue. While the existence and magnitude of the learning curve in LPD has been extensively explored, the proficiency threshold for RPD remains to be debated. Values as low as 7 RPD cases have been suggested, with other studies quoting more than 250 RPD cases as a threshold for attaining proficiency [11–14]. There is also a lack of consensus on which metric is the best descriptor of proficiency, with studies opting for reductions in operative time, estimated blood loss and morbidity as metrics of the proficiency threshold, without a standardised definition. A recent systematic review has identified heterogeneity in reporting of learning curve in open, laparoscopic and robotic pancreatic surgery, suggesting standardisation of definition of three learning phases (competency, proficiency, mastery) [15].

The learning curve effect has implications not only for trials reporting on RPD but also for future training of hepatopancreatobiliary surgeons. As such, understanding the factors that contribute to the learning effect and identifying the proficiency threshold is paramount to safe and widespread adoption of RPD, as well as assessment of its outcomes. Therefore, we analysed consecutive cases of RPD performed to date at our institution and aimed to assess the influence of procedural volume on short-term RPD morbidity outcomes, and operative variables. Additionally, we aimed at identifying a threshold of proficiency for RPD, expressed as a number of cases needed to overcome the learning curve.

## Methods

### Study setting

A prospectively maintained database of patients who underwent RPD was retrospectively reviewed to include all the cases of RPD between May 2017 (first RPD case) and December 2021 performed at our institution, a hepatopancreatobiliary tertiary referral centre in the United Kingdom. Our institution consists of two pancreatic surgeons who perform sixty pancreatic resections annually (40 PD and 20 distal pancreatectomies). One surgeon performs RPD whenever a robotic theatre is available. Prior to starting RPD this surgeon had performed 100 laparoscopic PD after a 10 year experience with open PD. Consecutive patients undergoing RPD for both malignant and benign pathologies were included in this analysis. RPD was performed with the Da Vinci Si, X and Xi models (Intuitive Surgical, California, USA) by a single senior surgeon (LJR). All patients selected for operative management following a multi-disciplinary discussion were considered for the robotic technique, but those with borderline resectable disease with the potential for venous invasion (portal vein (PV) or superior mesenteric vein (SMV)) identified on pre-operative imaging were excluded. At the time of this cohort (2017–2021) our institution did not give neoadjuvant chemotherapy (NAC) to patients with upfront resectable pancreatic ductal adenocarcinoma, while from 2020, those with borderline resectable disease were given NAC however these patients, likely to require vascular resection were scheduled for open PD rather than RPD. Cases which were abandoned intraoperatively were removed from the analysis. There was no cutoff with regards to BMI for consideration of robotic technique. The selection criteria for RPD did not change throughout the study period. This research followed the principles outlines in the Declaration of Helsinki, and written consent was sought for patients for inclusion in the database. This study was conducted according to STROBE guidelines [16].

### Robotic pancreatoduodenectomy

Our robotic technique is adapted from that originally published by Giulianotti et al. and was previously described by our group in detail [17, 18]. In brief, a four-port technique is utilised, with two extra 12 mm assistant ports. Patients were positioned in the Lloyd Davies position with 15° reverse Trendelenburg and 15° left-side tilt of the surgical table. Pneumoperitoneum is established with a sub-umbilical Hassan technique, and this incision is extended to 5 cm and used for specimen extraction. Dissection is performed with both the robotic hook diathermy and vessel sealer. The gastroduodenal artery is ligated and transfixed with 3/0 prolene suture and clipped with large robotic hem-o-lok clips (Intuitive Surgical, California USA). A 60 mm Endo GIA stapler or Echelon Flex stapler (Ethicon, Bridgewater, USA) is used for gastric and jejunal transection after kocherisation of the duodenum. Dissection of the uncinate process of the pancreas from the SMV and PV is performed using hook diathermy with medium-large hem-o-lok clips to the inferior pancreatoduodenal artery and vein. To complete dissection along the mesopancreas crural tissue, the authors’ preference is to use multiple EchelonFlex vascular staplers along the longitudinal plane in line with the portal vein. The tissue is divided posteriorly to the PV/SMV after visualisation of the SMA to the left. This standardised technique at our centre gives us consistent and reproducible results whilst minimising blood loss intraoperatively and avoiding postoperative secondary haemorrhage from small branches off SMA. A level 3 SMA neural tissue dissection and total arterial divestment is not routine in our centre for PDAC resections without vascular involvement. A pancreatico-jejunostomy (PJ) is performed using a modified Blumgart technique whenever PD is visible, and an internal pancreatic stent (infant feeding tube) is used. We use internal stents placed into the pancreatic duct and bile duct. A pancreatogastrostomy (PG) with an invagination technique is performed if the pancreatic duct is not visualised. The hepatico-jejunostomy (HJ) is performed as a continuous posterior layer and interrupted anterior layer with 3.0 or 4.0 PDS sutures. The gastro-jejunostomy (GJ) is created at the posterior wall of the stomach on a loop of jejunum at least 50 cm distally to the biliary anastomosis with a 45 mm Endo GIA stapler and V-Loc suture (Medtronic, Watford UK). Finally, a jejuno-jejunostomy is performed with a 45 mm EndoGIA stapler at least 10 cm from the GJ to assist with biliary drainage. All patients spend at least 24 h in the intensive care unit before recovery on the surgical ward. We start clear fluids on post-operative day (POD) 1 and aim to build to free fluids on POD3 with full diet on POD5 as tolerated. The nasogastric tube is removed when output is < 300 ml in 24 h. Surgical drains remain in situ until output is haemoserous and < 50 ml per day. Intravenous antibiotics are given at induction and three post-operative doses for patients without preoperative biliary intervention and 5 days for those with biliary intervention with stenting. We aim for discharge once the patient is tolerating a full diet, able to mobilise independently and pain is controlled with oral analgesics only.

### Outcome measures

All cases were classified as either low-risk or high-risk based on classification proposed by Sanchez-Velazquez et al. [19] The risk of postoperative pancreatic fistula was classified according to ISGPS guidelines (type A—non-soft texture and main pancreatic duct (MPD) size > 3 mm, type B—non-soft texture and MPD size ≤ 3 mm, type C—soft texture and MPD size > 3 mm and type D—soft texture and MPD size ≤ 3 mm) [20]. Operative details, including operative time and robotic console time, estimated blood loss and perioperative transfusion requirement, were retrieved from the database of RPD cases performed at our institution. Histopathological details, including final diagnosis, lymph node yield, resection margin status (R0 defined according to the Royal College of Pathologists as tumour cells > 1 mm from the resection margin on microscopic examination), tumour size, and lymph node positivity, was retrieved from electronic healthcare records. Moreover, data from the postoperative period, including the total length of hospital stay, length of ITU stay, 90-day morbidity (classified according to the Clavien-Dindo scale [21]), rates of postoperative pancreatic fistula (classified according to ISGPS guidelines) [20], re-operation rate and 90-day mortality were collected. Where available, electronic health records were searched for any follow-up appointment records to determine long-term outcomes of patients included, however, no additional formal long-term follow-up apart from standard care was provided as part of the study.

### Statistical analysis

Continuous data were summarised as median (interquartile range (IQR)) with non-parametric data identified following visual evaluation of the distribution and Shapiro–Wilk assessment for normality. To identify the learning effect and potential proficiency threshold, a cumulative sum (CUSUM) analysis was performed. Due to the single institution character and number of cases included, as well as no formal recording of long-term follow-up, we decided to focus on transition between competence and proficiency stages, whilst the transition from proficiency to mastery stages was outside of the scope of this study [15]. CUSUM curves were plotted for the cumulative difference between the expected and observed outcomes (Y-axis) against the consecutive number of cases of RPD (X-axis). Expected outcomes were defined as the mean of the outcome values across the whole dataset. At each case number, the curve goes upwards if the outcome is worse than expected and downwards if the outcome is better than expected. An inverse relationship between case number and operative outcomes was hypothesised. Therefore, proficiency threshold was defined as the number of consecutively performed cases, following which a change in the volume-outcome relationship occurs. As an inverse relationship between case volume and outcomes was hypothesised, it was accepted that the proficiency threshold would coincide where the curve peaked and changed direction from upward to downwards. Following identification of the threshold, the clinical significance of this threshold was determined by comparing outcomes between before-threshold and after-threshold subgroups, using the Mann–Whitney U test and X^2^ test. The threshold of statistical significance was set at *p* < 0.05. Data were analysed and presented using R3.6.3 (R Foundation for Statistical Computing, Vienna, Austria).

## Results

### Patient data

Sixty-five patients underwent a robotic pancreatoduodenectomy between May 2017 and December 2021 at our institution. One patient who has had an additional spleen-preserving total pancreatectomy was excluded from the analysis. Of the 64 cases remaining, four (4.7%) cases were abandoned due to metastatic spread of disease, resulting in 60 RPD cases completed, and included in the final analysis. The majority of were standard robotic pancreatoduodenectomy (*n* = 53, 88.3%), while seven (11.7%) were pylorus preserving RPDs.

The median age was 63.5 (range 19–86), and the majority of patients were male (*n* = 37, 61.7%). The average BMI was 27.2 ± 5.7 kg/m^2^ (range 18.0–44.0). The median pre-operative ASA grade was 3 (IQR 2–3), and three patients (5.0%) had undergone previous abdominal surgery. None of this RPD cohort received NAC. 24 (40.0%) were classified as low-risk cases, while 36 (60.0%) were classified as high risk cases. According to ISGPS classification, pancreas texture and main pancreatic duct size intraoperative assessment 28.3% (*n* = 17) of cases were type A, 1.7% (*n* = 1) type B, 28.3% (*n* = 17) type C, and 36.7% type D (*n* = 22). Pancreatic texture data were not available for three cases (5.0%).

### Operative data

The median operative time was 360 min (IQR 302.25–442 min), while the median robotic console time was 317.5 min (IQR 270–413 min). Two (3.3%) operations required conversion to open. One patient required perioperative transfusion related to intraoperative blood loss (1.7%), with an additional single patient (1.7%) having a clinically significant estimated blood loss of more than 500 ml.

### Post-operative data

Full post-operative outcomes data are present in Table 1. The median length of stay was 11 days (IQR 9–15), with a median ITU stay of 1 day (IQR 1–3 days). The total 90-day morbidity with a Clavien-Dindo complication of three or more was 25.0% (*n* = 15), while 34 patients had at least 1 complication (56.7%). The CR-POPF rate was 0%, with 6 (10.0%) patients developing biochemical leak. The re-operation rate was 6.7% (*n* = 4), in all cases due to post-operative bleeding, while endoscopic re-intervention occurred in one patient (1.7%) for bleeding gastric ulcer management. Two patients (3.3%) required interventional radiology embolisation for bleeding, while four patients (6.7%) required percutaneous drainage of collections. Delayed gastric emptying rate was 1.7% (*n* = 1). The overall 90-day mortality rate in the cohort was 1.7% (*n* = 1); this patient had a myocardial infarction on postoperative day seven.

**Table 1:** Postoperative outcomes data

Length of stay, days (median [IQR])	11 [9–15]
Length of ITU stay, days (median [IQR])	1 [1–3]
Morbidity, *n* (%)	
None	27 (45.0)
CD 1	5 (8.3)
CD 2	11 (18.3)
CD 3	12 (20.0)
CD 4	4 (6.7)
CD 5	1 (1.7)
POPF, *n* (%)	6 (10.0)
Biochemical leak, *n* (%)	6 (10.0)
Grade B, *n* (%)	0 (0.0)
Grade C, *n* (%)	0 (0.0)
Bile leak, *n* (%)	3 (5.0)
Delayed gastric emptying, *n* (%)	1 (1.7)
Endoscopic re-intervention, *n* (%)	1 (1.7)
Percutaneous drainage, *n* (%)	4 (6.7)
IR embolisation, *n* (%)	2 (3.3)
Re-operation, *n* (%)	2 (3.3)
90-day mortality, *n* (%)	1 (1.7)

*IQR* interquartile range, *ITU* intensive therapy unit, *POPF* Post-operative pancreatic fistula

### Histology

Histopathological data are summarised in Table 2. Most resected tumours were malignant (*n* = 46, 76.67), with pancreatic ductal adenocarcinoma being the most common tumour type, accounting for a third of all resected tumours (*n* = 20, 33.3%). For malignant tumours, the R0 resection rate was 71.2% (*n* = 33). The median number of lymph nodes (*n* = 20) retrieved was higher than the 15, recommended by The European Society for Medical Oncology (ESMO) [22].

**Table 2:** Histology data

**Histology, *n* (%)**	
Benign	14 (23.3)
IMPN	7 (11.6)
Duodenal Polyp	2 (3.3)
MCN	3 (5.0)
PanIN	1 (1.7)
Solid pseudopapillary tumour	1 (1.7)
Malignant	46 (76.7)
PDAC	20 (33.3)
Ampullary Ca	10 (16.7)
CCA	10 (16.7)
Duodenal Ca	2 (3.3)
NET	4 (6.7)
T stage, *n* (%)	
T0	1 (2.2)
T1	12 (26.1)
T2	9 (18.4)
T3	19 (41.3)
T4	4 (8.7)
N stage, *n* (%)	
N0	21 (45.6)
N1	11 (23.9)
N2	13 (28.2)
M stage, *n* (%)	
M0	45 (97.8)
M1	1 (2.2)
Resection margin, *n* (%)	
R0	33 (71.7)
R1	13 (28.2)
Tumour size, mm (mean ± SD, [range])	26.0 ± 13.6 [5–62]
No of retrieved lymph nodes (median [IQR])	20 [15.5–23.5]

*IPMN* Intraductal papillary mucinous neoplasm, *MCN* Mucinous cystic neoplasm, *PDAC* Pancreatic ductal adenocarcinoma, *CCA* Cholangiocarcinoma, *NET* Neuroendocrine tumour, *SD* standard deviation, *IQR* Interquartile range

### Long-term follow-up

Long-term follow-up data were available, with a mean follow-up of 8.8 months. During the study period, seven patients have died, resulting in an 88.3% overall survival rate in the cohort and 83.7% overall survival in those with malignant disease. 13.0% of those with a malignant pathology (*n* = 6) had evidence of disease recurrence within the follow-up period.

### Learning curve—operative time

**Fig. 1:**
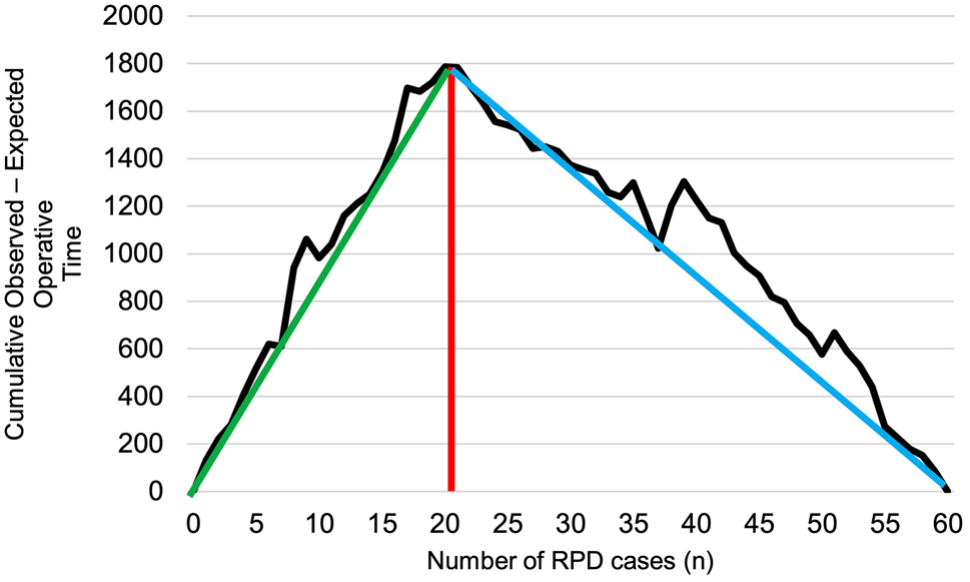
The learning curve for operative time. The red dotted line represents the inflexion point of the curve, which suggest the threshold of technical proficiency. *RPD* Robotic pancreatoduodenectomy. The first phase has a positive slope and is marked in green (learning phase), while the second phase has a negative slope and is marked in blue (proficiency phase)

CUSUM analysis has identified 21 cases as the threshold of proficiency, based on total operative time. The threshold was established according to the change in the inflexion of the curve from upward to downward (Fig. 1).

### Learning curve—all morbidity

**Fig. 2:**
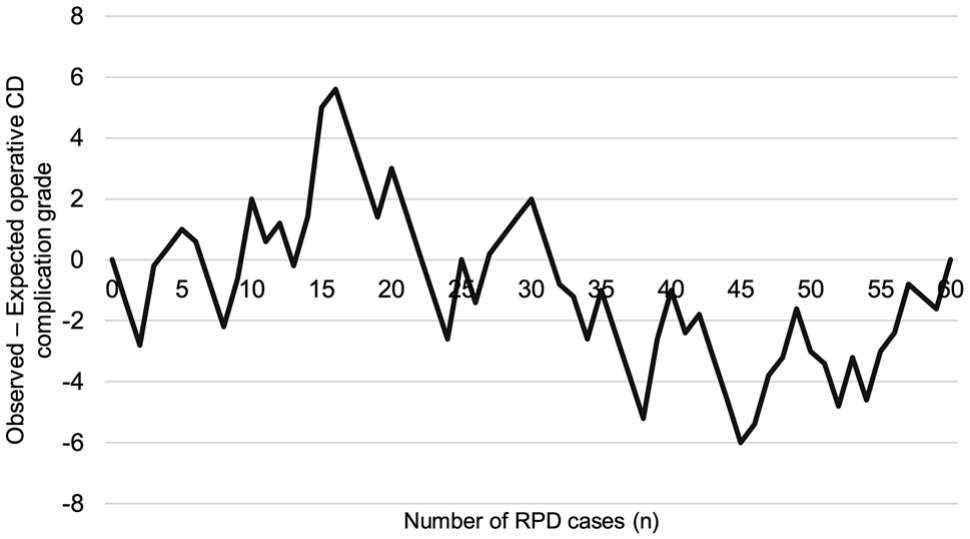
The learning curve for all morbidity. CUSUM calculated based on average CD grade. No clear inflexion point was identified on the curve. *CD* Clavien-Dindo, *RPD* Robotic pancreatoduodenectomy

Based on the CUSUM analysis of all grades of morbidity (minor and major), POPF rate, bile leak rate and re-operation rate, no clear threshold of procedures suggesting proficiency could not be identified (Fig. 2). Multiple additional inflexions of the curve were identified following the change in slope, and as such, no definitive number of cases could be derived.

### Learning phases

According to the proficiency threshold identified through operative time CUSUM analysis, the RPD outcomes were divided into the competency phase (cases 1–21) and proficiency phase (cases 22–60), following which the outcomes were compared in two subgroups. The subgroups were comparable in terms of age, sex, % of low-risk patients, % of PDAC patients, ASA grade and BMI, although of note, the competency phase subgroup had significantly no patients with previous abdominal surgery, although the difference was not significant 0% (*n* = 0) vs 7.7 (*n* = 3, *p* = 0.0598). A full comparison of baseline characteristics between the subgroups is presented in Supplementary Table 1. The operative time was significantly longer in the competency phase, compared with the proficiency phase (median 470 vs 320 min, *p* < 0.001). There were no significant differences in morbidity between the two subgroups in terms of all morbidity, major morbidity, biochemical leak and re-operation rates. A full comparison of operative outcomes is presented in Table 3.

**Table 3:** Operative outcomes stratified by proficiency threshold (*n* = 21) RPD cases

Outcomes	1–21 RPD procedures	22–60 RPD procedures	*p*-value
Operative time, min (median [IQR])	470 [420–500]	320 [299.5–360]	< 0.001
Length of stay, days (median [IQR])	13 [10–16]	11 [9–14]	0.165
Morbidity, median CD grade [IQR]	0 [1–3]	0 [1–3]	0.942
Major CD complications, *n* (%)	5 (23.8)	10 (25.6)	0.876
Biochemical leak, *n* (%)	2 (9.5)	4 (10.3)	0.928
Re-operation, *n* (%)	2 (9.5)	2 (5.1)	0.515
In-hospital or 30-day mortality, *n* (%)	1 (4.8)	0 (0.0)	N/A

*RPD* Robotic pancreatoduodenectomy, *IQR* interquartile range, *POPF* post-operative pancreatic fistula, *CD* Clavien-Dindo

## Discussion

Twenty-one cases of RPD were identified as technical proficiency threshold, indicated by curve inflexion on CUSUM analysis, and significant decrease in median operative time before and after the 21st case. However, the before-threshold and after-threshold groups did not differ in overall 90-day morbidity, major 90-day morbidity, biochemical leak rates, re-operation rates, and 90-day mortality.

The recent Miami International Evidence-Based Guidelines on Minimally Invasive Pancreas Resection has concluded that surgeon volume is associated with outcomes for minimally invasive PD, however, the quality of the existing evidence is weak [23]. The number of RPD procedures needed for reaching proficiency varies from 7 to 250 in the existing studies, depending on the outcomes used for constructing the learning phase curves [13, 14, 24]. Interestingly, Shi Y et al. have found a very similar threshold of proficiency (20 RPD cases) as the one in our series, with important consideration of previous robotic distal pancreatectomy experience needed before starting to perform RPDs [24]. Similarly, in our series, the surgeon performing the RPDs had some robotic distal pancreatectomy operative experience. Of note, our team had significant experience (up to 100 cases) performing laparoscopic PD and open PD prior to the introduction of RPD. This is likely to reflect a reduced operative learning curve compared to surgeons who only perform open PD prior to initiating robotic surgery.

No consensus has been reached about the optimal metric of proficiency [15, 23]. While some studies opt for operative time and estimated blood loss as measures of technical proficiency and competency, as we have elected to do in this analysis, morbidity and mortality outcomes might be a more appropriate choice [15]. However, we have found no significant differences in the occurrence of complications between the learning and proficiency phases. This may be attributed to our extensive procedural experience with both open PD and laparoscopic PD, as well as confirming that a robotic platform is suitable for performing complex procedures. It is also worth noting that while there were no significant differences in terms of age, sex, ASA and BMI, and % of cases being PDAC, % of cases being low-risk, and % of cases having had previous abdominal surgery, the learning phase had zero patients with previous abdominal operations, which could have influenced the total surgical time. However, such difference, if existing, would increase operating time in proficiency phase subgroup, due to additional time devoted to adhesiolysis, thus not affecting the main findings of the study. The % of low-risk cases was higher in proficiency group (48.7 vs 23.8%, *p* = 0.0603). While the indications for RPD did not change through study period, this being a non-comparative, non-randomised study, selection bias could have influenced the results, and differences between baseline characteristics of subgroups should be explored in more detail in the future to try to identify a subgroup of patients most likely to benefit from RPD.

The decrease in operative time after 21 cases is multifactorial. During the learning phase, prior to a standardised order of steps and technique, frequent changes of instruments and operative steps could have contributed to lengthening the operation. For example, rather than dissecting, clipping or tying and dividing tissues and pedicles individually we have now standardized the procedure to perform all the relevant dissections first in one particular area followed by clipping/tying and division in order to reduce the frequency of instrument changes. Further, the economy of motion, using the camera and three instrument arms, rapidly improves with continued practice. We hypothesise that during the competency phase, lack of standardisation of operative step sequence, combined with adaptation to new instruments and tasks, leads to increased operative time. The prior extensive experience in both open and laparoscopic pancreatic surgery in our centre could also have shortened the learning curve, however, the extent to which proficiently in the same surgical procedure across multiple surgical approaches (open vs laparoscopic vs robotic) is transferable has yet to be studied in detail. It is worth noting, that there was no difference in morbidity and POPF rates, suggesting that despite the existence of a technical proficiency curve, there was no short-term outcome learning curve effect present for RPD in our series [15]. When compared to international benchmark cohort, our case series, operative time, transfusion percentage, hospital stay, total morbidity, major CD morbidity (grades ≥ 3), POPF rates, biochemical leak, R1 rates, and number of lymph nodes resected were all below the benchmark cutoff [19]. Mortality in our cohort was 1.7%, while it was suggested cutoff of 1.6% should be utilised, while Grade 4 CD complication rates was 6.7%, while the suggested cutoff was 5%. Similar to the benchmark cohort study, our series featured 40% of low-risk, and 60% of high-risk cases (compared to 38% low-risk and 62% high-risk). Since long-term oncological data were not available at the end of study period for all patients, we did not perform benchmarking for those outcomes.

The benchmarking for operative variables attests to the robustness of outcomes in our case series. Of note, CR-POPF (0.0%) and re-operation (*n* = 4, 6.7%) rates were low across the cohort and comparatively lower than the rates reported in the existing literature [25] The conversion rate of 3.3% is also significantly improved from our previously published LPD cohort, which had a conversion rate of 24% [18]. We hypothesise that these outcomes are due to extensive previous experience in LPD, prior to starting an RPD programme at our institution, suggesting that there is an overlap between the learning curves between minimally invasive surgery platforms, despite the unique characteristics of RPD. Additionally, to performing LPD before RPD, the senior surgeon had performed parts of the RPD procedure robotically prior to conducting a full RPD. Indeed, this highlights the individualised nature of each surgeon’s learning curve, influenced by previous operative experience, suggesting a one-size fits-all approach should not be adopted. Further, we use an internal pancreatic stent (infant feeding tube) which we believe reduces our CR-POPF rate.

This study was based on a prospectively maintained database that encompasses all robotic procedures performed at our institution. Supplementing the database with additional data points obtained from a retrospective review of electronic healthcare records allowed for high data completeness and quality assurance. What is more, the pre-threshold and post-threshold groups were balanced in terms of pre-operative and histological factors, allowing for robust comparison between the learning and proficiency phases.

This study has limitations. It is based on a single-centre and single-surgeon operative experience, and as such, caution needs to be taken when applying conclusions derived from this cohort into other settings. As such, previous operative experience of the lead surgeon, case selection, and level of trainee involvement in the cases could have all influenced the number of cases needed to achieve technical proficiency and should be taken into consideration when generalising the results of the study and applying them to different settings. It is also worth noting, that in this study we focused on transition from competency to proficiency phase, and thus opted to focus on outcomes related to the technical aspects such as short-term complications and operative time. The transition from competency phase to mastery phase, which is arguably more important to long-term patient outcomes was not explored, due to the small sample size in this study, and lack of standardised follow up. Operative time and complication grade are only two of the possible metrics to assess proficiency, and later mastery in RPD. Other metrics such as estimated blood loss, CR-POPF rates, and long-term DFS and PFS rates could be used to assess phases of proficiency, especially in larger sample size, and multicentre studies. Moreover, while this study focused on RPD, a comparator group in the form of matched LPD or OPD cohorts would allow for a more in-depth analysis of the learning effect and proficiency threshold. We have also utilised three different DaVinci surgical robots across the series (Si, X and Xi), however, we have found little difference in terms of docking time between Si and X da Vinci with a head-on docking and Xi with side docking for PD, as there was no change in operative field required, hence no redocking intraoperatively which features in robotic colorectal resection. Finally, the senior surgeon (LRJ), who performed all of the RPD in this series, had previous experience in laparoscopic surgery and other robotic hepatopancreatobiliary procedures, including distal pancreatectomy, but had no procedural-specific robotic training or mentorship programme. Such mentorship programmes have been previously shown to the improve learning curve profiles and reduce the number of cases needed for proficiency, potentially allowing surgeons with no extensive prior PD experience to attain proficiency in a similar timeframe [26]. As such, for institutions looking to start RPD programmes, we would recommend following the three-phase model suggested by Hanye et al.—the first phase focusing on developing basic skills and procedure-specific skills with simulation, biotissue drills, video libraries, live case observations, and training courses, the second phase consisting of index procedures, fellowships, and proctoring programmes, and the third phase being the implementation of the procedure into standard practice [27]. Such an approach, although length in time, would aim to minimise the learning curve-associated morbidity and mortality, while putting emphasis on patient safety. This is now the recommended training by The Miami International Evidence-based Guidelines on minimally invasive pancreas resection.

The existence of the learning phase should have an impact on future clinical trials evaluating RPD against other surgical interventions. One of the early criticisms of minimally invasive PD is that it has higher complication rates and mortality compared with OPD, especially in low volume centres (< 10 minimally invasive PD per year) [28]. Establishing a minimal threshold for recruitment of centres and surgeons into future multicentre trials is, therefore, necessary to ensure that initial learning phase outcomes are not confounding the overall results. Indeed, the LEOPARD-2 trial, which aimed at comparing OPD with LPD has set a minimum of 20 LPD for any centre to enrol [29]. However, despite setting the threshold of 20 LPDs, that group was found to be associated with higher complication rates, raising concerns about using a one-size-fits-all approach to learning curves and suggesting a potentially higher number of cases needed for proficiency in LPD than in RPD [8]. The learning phase is shorter in robotic surgery compared with laparoscopic surgery, which will play an important role in surgical training as minimally invasive technique adoption increases [10]. While technical proficiency curves signify that RPD can be implemented safely and is technically feasible for a variety of benign and malignant indications, formal analyses in terms of long-term survival and oncological outcomes are needed in the future to establish the long-term benefit of RPD, and to determine if oncological outcomes improve over time, as number of cases performed increase [15].

As such, RPD should remain only confined to specialist centres with hepatopancreatobiliary experience and where a standardised robotic training program has been followed. In this way, RPD is safe to implement, as seen in the Netherlands and in Japan [30, 31]. A threshold of 21 cases for reaching technical proficiency has been found in this analysis, yet other factors should be considered for safe implementation of RPD, as learning curves are likely to be a surgeon- and institution-specific. What is more, the learning process likely does not stop with reaching proficiency, and more cases are needed to achieve mastery, which could be reflected in improvements in long-term, oncological outcomes [15]. In the future, single-surgeon outcomes analytics could be a useful tool for assessing competency ascertainment during training and quality control, while identifying phases of proficiency can allow for more targeted training and supervision. Future studies on RPD should focus on ensuring surgeons have reached proficiency in RPD to allow for objective outcome comparisons between RPD, LPD and OPD in order to elucidate the true effect of minimally invasive techniques on PD outcomes in the short and long-term.

## Supplementary Information

Below is the link to the electronic supplementary material.

Supplementary file1 (DOCX 14 KB)
