# Supplementary material for: The influence of procedural volume on short-term outcomes for robotic pancreatoduodenectomy—a cohort study and a learning curve analysis
Source: Surg Endosc. 2023 Mar 8;37(6):4719–27. doi: 10.1007/s00464-023-09941-8 (PMC10234850; doi:10.1007/s00464-023-09941-8)
Supplement: Supplementary file 1 — Supplementary file1 (DOCX 14 KB) [file 464_2023_9941_MOESM1_ESM.docx]

|  |  |  |  |
| --- | --- | --- | --- |
| **Baseline characteristics** | **Learning phase (n=21)** | **Proficiency phase (n= 39)** | **p-value** |
| Age (median, range) | 64 (19 – 84) | 63 (29 – 86) | 0.726 |
| Sex (Male, %) | 13 (61.9) | 24 (61.5) | 0.978 |
| BMI (mean±SD) | 27.0±5.7 | 27.4±5.7 | 0.835 |
| ASA grade (median, IQR) | 3 (3 – 3) | 2 (2 – 3) | 0.0598 |
| Previous abdominal surgery (Yes, %) | 0 (0) | 3 (7.7) | 0.201 |
| Low risk (Yes, %) | 5 (23.8) | 19 (48.7) | 0.0603 |
| PDAC (Yes, %) | 7 (33.3) | 13 (33.3) | 1.00 |

***Supplementary Table 1*** *– Baseline cohort characteristics after stratification into learning phase (n=21) and proficiency phase (n=39) subgroups. BMI – Body Mass Index. IQR – interquartile range. SD – Standard deviation. PDAC – Pancreatic Ductal Adenocarcinoma.*
